# Influence of acute or chronic calcium channel antagonists on the acquisition and consolidation of memory and nicotine-induced cognitive effects in mice

**DOI:** 10.1007/s00210-013-0866-z

**Published:** 2013-04-12

**Authors:** Grazyna Biala, Marta Kruk-Slomka, Krzysztof Jozwiak

**Affiliations:** 1 Department of Pharmacology with Pharmacodynamics, Medical University of Lublin, 4A Chodzki str, 20-093 Lublin, Poland; 2 Laboratory of Medicinal Chemistry and Neuroengineering, Medical University of Lublin, Lublin, Poland

**Keywords:** Memory, Calcium channel antagonists, Nicotine, Cholinergic receptors, Modified elevated plus maze, Mice

## Abstract

Nicotinic cholinergic receptors (nAChRs) form a heterogeneous family of ligand-gated ion channels found in the nervous system. The main objective of our research was to investigate the interaction between cholinergic nicotinic system and calcium homeostasis in cognitive processes using the modified elevated plus maze memory model in mice. The time each mouse took to move from the open arm to either of the enclosed arms on the retention trial (transfer latency, TL2) was used as an index of memory. Our results showed that a single injection of nicotine (0.035 and 0.175 mg/kg) shortened TL2 values, improving memory-related processes. Similarly, L-type calcium channel antagonists (CCAs), i.e., flunarizine, verapamil, amlodipine, nimodipine, nifedipine, and nicardipine (at the range of dose 5–20 mg/kg) administered before or after training, decreased TL2 value improving memory acquisition and/or consolidation. Interestingly, at the subthresold doses, flunarizine, nicardipine, amlodipine, verapamil, and bupropion, a nAChR antagonist, significantly reversed the nicotine improvement of memory acquisition, while flunarizine, verapamil, and bupropion attenuated the improvement of memory consolidation provoked by an acute injection of nicotine (0.035 mg/kg, s.c.). After subchronic administration (14 days, i.p.) of verapamil and amlodipine, two CCAs with the highest affinity for nAChRs, only verapamil (5 mg/kg) impaired memory acquisition and consolidation while both verapamil and amlodipine, at the subthresold, ineffective dose (2.5 mg/kg), significantly reversed the improvement of memory provoked by an acute injection of nicotine (0.035 mg/kg, s.c.). Our findings can be useful to better understand the interaction between cholinergic nicotinic receptors and calcium-related mechanisms in memory-related processes.

## Introduction

Neuronal nicotinic cholinergic receptors (nAChRs) form a heterogeneous family of ligand-gated ion channels found in the central and peripheral nervous system that regulate synaptic activity (Jackson et al. 2008; Picciotto et al. 2000; Stolerman and Shoaib 1991; Wonnacott 1997). Numerous subtypes of nAChRs have been identified and many of them were recognized to be involved in specific neurological and physiological behaviors. For instance, α3β2 nAChR plays a role in dopamine release and Parkinson’s disease, α3β4 regulates noradrenaline release and cardiovascular or gastrointestinal action, and α9 was found important in development of auditory functions. Moreover, the most abundant subtypes of the nAChRs in the cortex, i.e., α4β2, α4β4, and α7 are involved in memory, learning, and sensory gating functions (Gotti et al. 2006).

nAChRs are activated by endogenous acetylcholine (ACh) and the group of ortosteric agonists, such as nicotine, while their activity is inhibited by a diverse group of competitive antagonists. Except for these actions, different subtypes of nAChR can be modulated allosterically by various endogenous [e.g., substance P, serotonin (5-HT), fatty acids, steroids or β-amyloid] as well as exogenous (e.g., alkaloids, venom toxins, alcohol, and other drugs) substances with different binding sites on the nAChRs (Moaddel et al. 2007). In fact, over 50 marketed drugs belonging to different therapeutic classes exert allosteric positive (noncompetitive agonists) or allosteric negative (noncompetitive antagonists) modulation on nAChRs, and many of these actions are subtype specific. For a long time, these off-target interactions did not attract significant recognition up to recently when modulation of nAChRs is being linked to specific adverse effects observed during certain therapies (Friederich et al. 2000). For example, constipation induced by verapamil or methadone is regarded to be a result of strong inhibition of specific subtypes of nAChRs.

The main nAChRs serve as potential therapeutic targets for a many different diseases (Bencherif and Schmitt 2002; Buccafusco 2004). For example, nicotine and other nAChR agonists with differential subtype selectivity have been identified as potential cognition-enhancing therapeutic drugs, particularly for the treatment of Alzheimer’s disease (AD) (Bencherif and Schmitt 2002; Buccafusco 2004; Levin 2002; Moaddel et al. 2007; Picciotto and Zoli 2002). It has been commonly accepted that the progressive loss of cholinergic neurons is one of the cornerstone of AD pathology, and the association between nAChR and cognitive decline in AD has been widely investigated. For the past several years, a mainstay of the AD therapy has been aimed at inhibiting acetylcholinesterase and thereby increasing ACh levels in the central nervous system. Three such inhibitors, donepezil, galantamine, and rivastigmine, are now in clinical practice for treatment of mild and moderate AD. Currently, selective activation of α4β2 and/or α7 nAChRs is also under investigation as a therapeutic strategy in AD treatment and several such agonists are in clinical trials (Moaddel et al. 2007).

As already mentioned, nicotine itself shows neuroprotective properties and several epidemiological studies claim a lower incidence of AD in tobacco habitual smokers; the latter statement, however, is in conflict with some other more recent reports (Levin 2002; Picciotto and Zoli 2002; Sabbagh et al. 2002). All of them suggest that exogenous modulation of the subtypes of nAChRs, especially in the longer time frame, has an impact on cognitive functions in elderly people and development of AD symptoms. Additionally, studies on several aging populations indicate the higher risk of dementia and/or AD in hypertension patients treated with certain calcium channel antagonists (CCAs) comparing to those treated with other hypotensive drugs (Maxwell et al. 1999; Khachaturian et al. 2006). Accordingly, it has been recently suggested that many commonly used CCAs can be strong noncompetitive inhibitors for the α3β4 subtype of nAChRs (Jozwiak et al. 2005, 2008). Several clinical and experimental studies have already evaluated the potential of antihypertensive medications for modification of the risk of AD. In this large population-based study of persons aged 65 years and older, the authors found reduced risks of AD among subjects using specific types of medications (Khachaturian et al. 2006). Suggestive trends were observed with beta-blockers and dihydropyridine CCAs independently of their ability to control blood pressure. On the other hand, concern has been raised about the potential for adverse cognitive effects associated with the use of CCAs in older people who, according to other author, were significantly more likely than those using other agents to experience cognitive decline (Khachaturian et al. 2006, for review).

Considering the results mentioned above supported by evidence from epidemiological studies, we intended to further investigate the effect of nicotine on memory-related behavior as well as the interaction between cholinergic nicotinic system and calcium homeostasis in cognitive processes using the recently developed modified elevated plus maze (mEPM) memory model in mice (Biala and Kruk 2008; Itoh et al. 1990; Kruk et al. 2011; Kruk-Slomka et al. 2012). Recently, this test, originally developed to estimate anxiety in rodents, was modified to evaluate spatial learning and memory. Briefly, this simple method consists of measuring of the time necessary for the animal to move from the open to the enclosed arm, i.e., the transfer latency (TL). This protocol demonstrates that the information acquired in the first trial is consolidated within 24 h after the acquisition session and successfully recalled during the second trial. A reduction in TL using the retention trial followed 24 h represents an improvement in learning and memory and has been interpreted as the ability for animals to remember the location of the enclosed arms and escape from the unsafe open and high space faster on the second retention trial. On the contrary, increases in TL during retention testing could be used to indicate impairments in memory induced by drugs that possess amnesic properties. This method has been successfully used in studies investigating the involvement of different neurotransmitter systems, including cholinergic pathways, on learning and memory processes. For instance, amnesic properties of scopolamine, a muscarinic receptor antagonist, were evaluated. Indeed, scopolamine increased the TL time, while physostigmine decreased the TL values on the second retention trial and reversed the effects of scopolamine (Hliňák and Krejčí 1998, 2002; Itoh et al. 1991; Sharma and Kulkarni 1992).

Based on previous experiments in vitro (Jozwiak et al. 2005, 2008), the characterization of drug action was then performed to qualify substances for in vivo animal test. For this purpose, in continuation of our efforts to understand the neurobiology of memory and learning processes, we investigated the influence of acute and subchronic administration of CCAs on the acquisition and consolidation of memory-related processes as well as on the memory enhancing effects of nicotine. To this end, L-type voltage-dependent calcium channel (VDCC) antagonists of various classes including dihydropyridines (e.g., nimodipine, nifedipine, nicardipine, amlodipine), phenylalkylamines (e.g., verapamil), benzothiazepines (e.g., diltiazem), and diphenylalkylamines (e.g., flunarizine) were used. The range of doses and timing was selected taking into account studies of behavioral effects of VDCC blockers conducted on rodents and obtained previously in our laboratory (Biala 2003; Biala and Budzynska 2006, 2008; Biala and Weglinska 2004, 2005). Accordingly, at these doses, the drugs did not affect locomotor activity measured either in the EPM test or in the actimeter cages. We have chosen these different classes of compounds as the results, including those of our group already cited, indicate that various classes of calcium antagonists differ in their interaction with the effects of psychoactive substances as well with nAChR subtypes. In comparison with our previous study describing memory-related effects of CCAs and nicotine, in the present new and original study, larger range of doses of seven selected compounds was used, both acute and repeated CCAs injections and, importantly, both stages of memory, i.e., acquisition and consolidation also in the context of possible influence on nicotine cognitive effects, were studied. The following criteria were used to select compounds for these experiments: (a) drugs commonly used for chronic treatment, especially in cardiovascular disorders, (b) strong modulators of subtypes of receptor involved in AD development (α2β2, α2β4, and α7), and (c) drugs, for which epidemiological data suggest an increased (or decreased) risk of AD incidence in patients with the long term treatment history. Our study can be useful to better understand the common cholinergic/calcium-dependent mechanisms of memory formation and may provide new perspectives for the promising therapy of human disorders, in which cholinergic signaling has been implicated, including AD, dementia, and addiction.

## Materials and methods

### Animals

The experiments were carried out on naive male Swiss mice (Farm of Laboratory Animals, Warszawa, Poland), about 1 month old, weighing 20–30 g. The animals were maintained under standard laboratory conditions (12-h light/dark cycle; room temperature, 21 ± 1 °C; 40–50 % humidity) with free access to tap water and laboratory chow (Bacutil, Motycz, Poland) in their home cages and were adapted to the laboratory conditions for at least 1 week before the experiments. Each experimental group consisted of 8–15 animals. Before and during the experiments, animals were kept in group consisting of 10 animals. The total number of animals used in all experiments was about 930.

All behavioral experiments were performed between 0800 and 1400 hours and were conducted according to the National Institute of Health Guidelines for the Care and Use of Laboratory Animals and the European Community Council Directive for the Care and Use of Laboratory Animals of 24 November 1986 (86/609/EEC). All experiments were approved by the local ethics committee (license no. 25/2008).

### Drugs

The following compounds were tested: (−)-nicotine hydrogen tartrate (0.0175, 0.035, 0.175, or 0.35 mg/kg, reported in freebase nicotine weight; Sigma-Aldrich, St. Louis, MO, USA), bupropion hydrochloride (10, 20, and 40 mg/kg, Sigma-Aldrich, St. Louis, MO, USA), verapamil hydrochloride (2.5, 5, 10, or 20 mg/kg, Sigma-Aldrich, St. Louis, MO, USA), diltiazem hydrochloride (5, 10, or 20 mg/kg, Sigma-Aldrich, St. Louis, MO, USA), amlodipine besylate (2.5, 5, 10, or 20 mg/kg, Sigma-Aldrich, St. Louis, MO, USA), flunarizine dihydrochloride (5, 10, or 20 mg/kg, Sigma-Aldrich, St. Louis, MO, USA), nimodipine (5, 10, or 20 mg/kg, Sigma-Aldrich, St. Louis, MO, USA), nicardipine hydrochloride (2.5, 5, 10, or 20 mg/kg, Sigma-Aldrich, St. Louis, MO, USA), and nifedipine (5, 10, or 20 mg/kg, Sigma-Aldrich, St. Louis, MO, USA). All compounds were dissolved in saline solution (0.9 % NaCl). Except for nicotine, the drug doses refer to the salt form. The pH of the nicotine solution was adjusted to 7.0. Fresh drug solutions were prepared on each day of experimentation. All agents were administered subcutaneously (s.c.) or intraperitoneally (i.p.) at a volume of 10 ml/kg. Control groups received saline injections of the same volume and via the same route of administration.

### Apparatus and experimental procedures

Memory and learning responses were measured using the mEPM test as described previously (Biala and Kruk 2008; Kruk et al. 2011). The experimental apparatus was shaped like a “plus” sign and consisted of a central platform (5 × 5 cm), two open arms (5 × 30 cm), and two enclosed arms (5 × 30 × 15 cm) opposite to each other. The maze was made of dark Plexiglas. The whole apparatus was elevated 50 cm above the floor, kept in a soundproof room with a neutral masking noise and a dim 40 lx illumination.

In the mEPM test, the time that the mice took to move from the open arm to the enclosed arm was used as the index of learning and memory and defined as TL. The mice were placed individually at the end of the open arm, facing it away from the central platform. Each group was submitted to the same procedure twice (the interval between the trials was 24 h). On the first trial (acquisition trial), the time each mouse took to move from the open arm to either of the enclosed arms (transfer latency, TL1) was observed on a monitor through a video camera system and recorded by the experimenter blind to the experimental group. If the mice failed to enter the enclosed arm within 90 s, they were placed at an enclosed arm and permitted to explore the plus maze for additional 60 s. In such a case, the TL1 value was recorded as 90 s. On the next trial (retention trial), 24 h later, the test was performed in the same manner as the first trial and the TL was recorded (TL2). If the mouse did not enter the enclosed arm within 90 s, the test was stopped, and the TL2 was recorded as 90 s. Any animal that fell off the maze was excluded from the experiment. In the mEPM test, we used the TL2 values on the retention trial as an index of memory and learning effects. Entry into one arm was recorded when an animal placed all four paws past the line dividing the central square from the open arms. The test arena was wiped with a damp cloth after each trial.

The mEPM task allows investigating different stages of memory, depending on the time of drug treatment. Thus, administration of the drug before the first trial (before pretest) should interfere with the acquisition of information, while its administration immediately after the first trial (after pretest) should affect the processes of consolidation. In our experiments, the drugs were administered 30 min before the pretest or immediately after the pretest, and the effects of each compound on memory acquisition and consolidation were investigated.

### Treatment

The first experiment was designed to investigate the influence of either nicotine or CCAs on memory-related responses using the mEPM in mice. Nicotine (0.0175, 0.035, 0.175, and 0.35 mg/kg, s.c.), verapamil (2.5, 5, 10, and 20 mg/kg, i.p.), diltiazem (5, 10, and 20 mg/kg, i.p.), amlodipine (2.5, 5, 10, and 20 mg/kg, i.p.), flunarizine (5, 10, and 20 mg/kg, i.p.), nimodipine (5, 10, and 20 mg/kg, i.p.), nicardipine (2.5, 5, 10, and 20 mg/kg, i.p.), nifedipine (5, 10, and 20 mg/kg, i.p.), bupropion (10, 20, and 40 mg/kg), for comparison, or saline were administered 30 min before the first trial (memory acquisition) or immediately after the first trial (memory consolidation). Twenty-four hours later, the animals were retested in the mEPM test. This second day, the mice did not receive any injection.

The second set of experiments was designed to investigate the influence of acute CCAs administration, at the inactive doses, on the acquisition and consolidation of memory-related responses induced by acute nicotine administration. For this purpose, verapamil (2.5 mg/kg, i.p.), amlodipine (2.5 mg/kg, i.p.), flunarizine (5 mg/kg, i.p.), nicardipine (2.5 mg/kg, i.p.), and bupropion (10 and 20 mg/kg, i.p.) or saline were administered 15 min prior to effective dose of nicotine (0.035 mg/kg, s.c.), and then the mice were tested 30 min later and retested after 24 h (memory acquisition) in the mEPM test. Additional groups of mice were injected as described above, but immediately after the first trial. The animals were then retested after 24 h (memory consolidation) in the mEPM test.

In the next set of experiments, we evaluated the influence of chronic CCAs (i.e., verapamil and amlodipine) administration on the acquisition and consolidation of memory-related responses using the mEPM in mice. For this purpose, the mice were randomly allocated to receive 13 days of i.p. injections of verapamil (2.5 and 5 mg/kg), amlodipine (2.5 and 5 mg/kg), or saline (for the control group). After an additional injection on the 14th day, the mice were tested 30 min later and retested after 24 h (memory acquisition) in the mEPM test. The remaining group of mice were also injected with verapamil (2.5 and 5 mg/kg, i.p.), amlodipine (2.5 and 5 mg/kg, i.p.), or saline immediately after the first trial. These animals were then retested after 24 h (memory consolidation).

The last experiment was designed to examine the influence of above-mentioned chronic CCAs administration on the acquisition and consolidation of memory-related responses induced by an acute nicotine injection. For this purpose, verapamil (2.5 mg/kg, i.p.), amlodipine (2.5 mg/kg, i.p.), or saline were administered for 13 days. On the 14th day, some animals were subjected to verapamil (2.5 mg/kg, i.p.), amlodipine (2.5 mg/kg, i.p.), or saline, 15 min prior to acute nicotine (0.035 mg/kg, s.c.) administration. Then, the mice were tested 30 min later and retested after 24 h (memory acquisition) in the mEPM test. The remaining group of mice was injected as described above, but immediately after the first trial. These animals were then retested after 24 h (memory consolidation).

### Statistics

The data were expressed as the means ± SEM. For the mEPM test, we measured TL, i.e., the time necessary for the mice to move from the open arm to either of the enclosed arms. The statistical analyses were performed using the one-way analyses of variance (ANOVA). A post hoc comparison of means was carried out using Tukey’s test for multiple comparisons, when appropriate. The data were considered statistically significant at a confidence limit of *P* < 0.05.

## Results

Across all experiments, the time (in seconds) that each mouse took to move from the open arm to either of the enclosed arms on the first trial (pretest), i.e., TL1, did not significantly differ among groups. Moreover, nicotine, CCAs or bupropion administered before this first trial had no influence on TL1 values across experiments (Table 1).

**Table 1:** Effects of acute nicotine, bupropion, CCAs, or saline injection on the transfer latency to the enclosed arm on the first trial (TL1) using the mEPM test in mice

Compound	TL 1
Saline	59.00 ± 7.78
Nicotine 0.0175 mg/kg	47.80 ± 15.23
Nicotine 0.035 mg/kg	51.20 ± 10.21
Nicotine 0.175 mg/kg	45.10 ± 9.15
Nicotine 0.35 mg/kg	50.33 ± 4.35
Bupropion 10 mg/kg	43.10 ± 4.41
Bupropion 20 mg/kg	45.00 ± 2.51
Bupropion 40 mg/kg	50.10 ± 7.20
Amlodipine 2.5 mg/kg	43.60 ± 4.35
Amlodipine 5 mg/kg	52.60 ± 5.83
Amlodipine 10 mg/kg	36.40 ± 6.04
Amlodipine 20 mg/kg	36.80 ± 4.63
Flunarizine 5 mg/kg	48.33 ± 5.39
Flunarizine 10 mg/kg	51.20 ± 5.84
Flunarizine 20 mg/kg	42.93 ± 4.98
Nimodipine 5 mg/kg	41.50 ± 8.44
Nimodipine 10 mg/kg	49.20 ± 6.31
Nimodipine 20 mg/kg	42.50 ± 4.51
Nifedipine 5 mg/kg	42.20 ± 7.43
Nifedipine 10 mg/kg	47.40 ± 7.86
Nifedipine 20 mg/kg	44.80 ± 9.22
Nicardipine 2.5 mg/kg	49.13 ± 3.98
Nicardipine 5 mg/kg	52.50 ± 5.34
Nicardipine 10 mg/kg	53.60 ± 8.06
Nicardipine 20 mg/kg	49.20 ± 7.39
Verapamil 2.5 mg/kg	58.90 ± 7.61
Verapamil 5 mg/kg	52.50 ± 7.22
Verapamil 10 mg/kg	55.90 ± 4.69
Verapamil 20 mg/kg	44.67 ± 10.44
Diltiazem 5 mg/kg	55.31 ± 6.15
Diltiazem 10 mg/kg	48.80 ± 4.70
Diltiazem 20 mg/kg	47.75 ± 6.45

Nicotine, bupropion, CCAs, or saline were administered 30 min before the first trial; *n* = 8–15; the data are shown as the means ± SEM

### Influence of acute nicotine, CCAs, or bupropion administration on memory-related processes in the mEPM model in mice

One-way ANOVA revealed that acute s.c. doses of nicotine (0.0175, 0.035, 0.175, or 0.35 mg/kg) and acute i.p. doses of CCAs used, i.e., amlodipine, flunarizine, nimodipine, nicardipine, verapamil, diltiazem (2.5–20 mg/kg), or bupropion (10–40 mg/kg) had a statistically significant effect on TL2 values [*F*(31,308) = 6.103; *P* < 0.0001], with respect to memory acquisition during the retention trial. Indeed, post hoc Tukey’s test revealed that the mice treated with nicotine (0.035 and 0.175 mg/kg), amlodipine (5, 10, and 20 mg/kg), nicardipine (5, 10, and 20 mg/kg), and flunarizine (20 mg/kg) significantly decreased TL2 values compared with saline-treated mice, indicating that these compounds improved memory and learning acquisition processes (*P* < 0.05 for nicardipine and flunarizine; *P* < 0.001 for nicotine and amlodipine; Table 2).

**Table 2:** Effects of acute nicotine, bupropion, CCAs, or saline injection on the transfer latency to the enclosed arm in the acquisition trial using the mEPM test in mice

Compound	TL2
Saline	33.60 ± 3.30
Nicotine 0.0175 mg/kg	25.30 ± 4.75
Nicotine 0.035 mg/kg	12.79 ± 1.14**
Nicotine 0.175 mg/kg	11.75 ± 2.08**
Nicotine 0.35 mg/kg	25.50 ± 5.35
Bupropion 10 mg/kg	51.75 ± 6.29
Bupropion 20 mg/kg	38.70 ± 4.94
Bupropion 40 mg/kg	41.20 ± 8.72
Amlodipine 2.5 mg/kg	29.60 ± 4.41
Amlodipine 5 mg/kg	15.10 ± 1.71**
Amlodipine 10 mg/kg	11.40 ± 1.28**
Amlodipine 20 mg/kg	7.20 ± 1.06**
Flunarizine 5 mg/kg	22.67 ± 6.12
Flunarizine 10 mg/kg	28.07 ± 4.38
Flunarizine 20 mg/kg	14.71 ± 2.50*
Nimodipine 5 mg/kg	39.80 ± 2.45
Nimodipine 10 mg/kg	30.60 ± 3.02
Nimodipine 20 mg/kg	25.10 ± 3.83
Nifedipine 5 mg/kg	26.30 ± 5.08
Nifedipine 10 mg/kg	23.00 ± 2.67
Nifedipine 20 mg/kg	26.70 ± 3.61
Nicardipine 2.5 mg/kg	29.50 ± 5.57
Nicardipine 5 mg/kg	22.20 ± 3.31*
Nicardipine 10 mg/kg	22.40 ± 2.19*
Nicardipine 20 mg/kg	22.10 ± 2.45*
Verapamil 2.5 mg/kg	45.00 ± 6.03
Verapamil 5 mg/kg	29.90 ± 4.44
Verapamil 10 mg/kg	31.50 ± 4.27
Verapamil 20 mg/kg	20.67 ± 3.68
Diltiazem 5 mg/kg	16.69 ± 3.49
Diltiazem 10 mg/kg	30.40 ± 4.00
Diltiazem 20 mg/kg	49.75 ± 7.83

Nicotine, bupropion, CCAs, or saline were administered 30 min before the first trial; *n* = 8–15; the data are shown as the means ± SEM

*P < 0.05; **P < 0.001 vs. saline-treated group (Tukey’s test)

Similarly, for memory consolidation during the retention trial, the mice receiving acute s.c. doses of nicotine (0.0175, 0.035, 0.175, or 0.35 mg/kg) and acute i.p. doses of CCAs used, i.e., amlodipine, flunarizine, nimodipine, nicardipine, verapamil, diltiazem (2.5–20 mg/kg), or bupropion (10–40 mg/kg) had a statistically significant effect on TL2 values [*F*(31,308) = 4.660; *P* < 0.0001]. Indeed, post hoc Tukey’s test revealed that the mice treated with nicotine (0.035 and 0.175 mg/kg), amlodipine (5, 10, and 20 mg/kg), nimodipine (20 mg/kg), nifedipine (10 and 20 mg/kg), and verapamil (5 and 10 mg/kg) significantly decreased TL2 values compared with saline-treated mice, indicating that these compounds improved memory and learning consolidation processes (*P* < 0.05 for nicotine and amlodipine 10 mg/kg; *P* < 0.01 for nimodipine, nifedipine, verapamil, and amlodipine 5 and 20 mg/kg; Table 3).

**Table 3:** Effects of an acute nicotine, bupropion, CCAs, or saline injection on the transfer latency to the enclosed arm in the consolidation trial using the mEPM test in mice

Compound	TL2
Saline	36.50 ± 9.08
Nicotine 0.0175 mg/kg	42.00 ± 5.12
Nicotine 0.035 mg/kg	17.36 ± 1.14*
Nicotine 0.175 mg/kg	13.86 ± 1.58*
Nicotine 0.35 mg/kg	23.50 ± 2.80
Bupropion 10 mg/kg	51.78 ± 5.39
Bupropion 20 mg/kg	46.00 ± 8.82
Bupropion 40 mg/kg	36.44 ± 5.36
Amlodipine 2.5 mg/kg	39.80 ± 7.60
Amlodipine 5 mg/kg	13.00 ± 1.13**
Amlodipine 10 mg/kg	14.70 ± 1.65*
Amlodipine 20 mg/kg	10.10 ± 0.88**
Flunarizine 5 mg/kg	25.80 ± 5.49
Flunarizine 10 mg/kg	20.73 ± 3.28
Flunarizine 20 mg/kg	24.93 ± 4.56
Nimodipine 5 mg/kg	36.10 ± 2.18
Nimodipine 10 mg/kg	28.00 ± 2.67
Nimodipine 20 mg/kg	16.50 ± 1.60**
Nifedipine 5 mg/kg	22.20 ± 3.48
Nifedipine 10 mg/kg	14.30 ± 3.22**
Nifedipine 20 mg/kg	14.60 ± 1.97**
Nicardipine 2.5 mg/kg	24.70 ± 9.27
Nicardipine 5 mg/kg	29.60 ± 7.35
Nicardipine 10 mg/kg	23.40 ± 3.31
Nicardipine 20 mg/kg	28.40 ± 7.27
Verapamil 2.5 mg/kg	32.50 ± 3.15
Verapamil 5 mg/kg	12.30 ± 1.22**
Verapamil 10 mg/kg	15.50 ± 2.45**
Verapamil 20 mg/kg	23.60 ± 3.61
Diltiazem 5 mg/kg	31.23 ± 5.71
Diltiazem 10 mg/kg	22.27 ± 2.08
Diltiazem 20 mg/kg	28.33 ± 4.98

Nicotine, bupropion, CCAs, or saline were administered immediately after the first trial; *n* = 8–15; the data are shown as the means ± SEM

*P < 0.05; **P < 0.01 vs. saline-treated group (Tukey’s test)

### Influence of acute CCAs or bupropion administration on memory-related responses induced by an acute nicotine using the mEPM model in mice

**Fig. 1:**
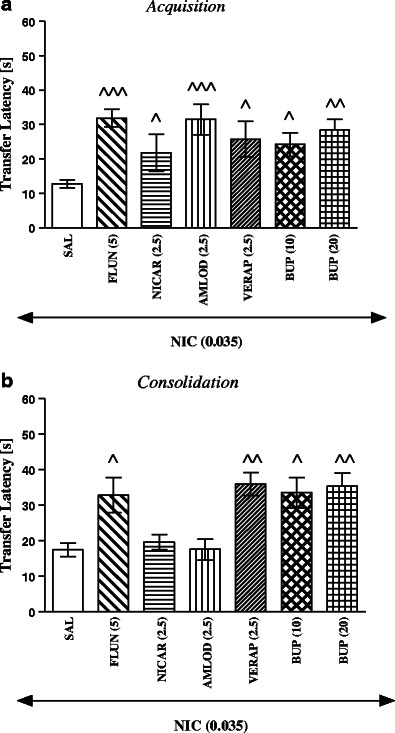
Influence of an acute CCAs or bupropion administration on the memory-related responses induced by an acute nicotine injection in the acquisition trial (**a**) or consolidation trial (**b**) using the mEPM test in mice. Flunarizine (5 mg/kg, i.p.), nicardipine (2.5 mg/kg, i.p.), amlodipine (2.5 mg/kg, i.p.), verapamil (2.5 mg/kg, i.p.), bupropion (10 and 20 mg/kg, i.p.), or saline were administered 15 min prior to saline or nicotine (0.035 mg/kg, s.c.) injection, 30 min before the first trial (**a**) or immediately after the first trial (**b**); *n* = 8–14; the data are shown as the means ± SEM; ^*P* < 0.05; ^^*P* < 0.01; ^^^*P* < 0.001 vs. nicotine-treated group (Tukey’s test)

Figure 1a shows the effect of pretraining of flunarizine (5 mg/kg), nicardipine (2.5 mg/kg), amlodipine (2.5 mg/kg), verapamil (2.5 mg/kg), or bupropion (10 and 20 mg/kg), i.p., on the improvement of memory acquisition induced by nicotine (0.035 mg/kg, s.c.) injection. One-way ANOVA revealed that there was a statistically significant effect [*F*(6,63) = 3.905, *P* = 0.0022], with respect to memory acquisition during the retention trial. In this experiment, flunarizine, nicardipine, amlodipine, verapamil, and bupropion, at the subthresold doses, significantly reversed the improvement of memory provoked by an acute injection of nicotine, thus resulting in an increased TL2 time compared to the nicotine-treated group (*P* < 0.05 for nicardipine, verapamil, and bupropion 10 mg/kg; *P* < 0.01 for bupropion 20 mg/kg; *P* < 0.001 for flunarizine and amlodipine, Tukey’s test; Fig. 1a).

Similarly, Fig. 1b shows the effect of pretreatment of flunarizine (5 mg/kg), nicardipine (2.5 mg/kg), amlodipine (2.5 mg/kg), verapamil (2.5 mg/kg), or bupropion (10 and 20 mg/kg), i.p., on the improvement of memory consolidation induced by nicotine (0.035 mg/kg, s.c.) injection. One-way ANOVA revealed that there was a statistically significant effect [*F*(6,63) = 7.223, *P* < 0.0001], with respect to memory consolidation during the retention trial. In this experiment, flunarizine, verapamil, and bupropion, but not nicardipine or amlodipine, at the subthresold doses, significantly reversed the improvement of memory provoked by an acute injection of nicotine, thus resulting in an increased TL2 time compared to the nicotine-treated group (*P* < 0.05 for flunarizine and bupropion 10 mg/kg; *P* < 0.01 for verapamil and bupropion 20 mg/kg, Tukey’s test; Fig. 1b).

### Influence of chronic CCAs administration on memory-related processes in the mEPM model in mice

One-way ANOVA revealed that chronic i.p. administration of verapamil (2.5 and 5 mg/kg) and amlodipine (2.5 and 5 mg/kg) had a statistically significant effect on TL2 values [*F*(4,35) = 2.902, *P* = 0.0357], with respect to memory acquisition during the retention trial. Indeed, post hoc Tukey’s test revealed that mice treated only with verapamil (5 mg/kg) significantly increased TL2 values compared with saline-treated mice, indicating that verapamil, at this dose used, impaired memory and learning processes after subchronic administration (*P* < 0.05; Table 4). Similarly, for memory consolidation during the retention trial, the mice receiving chronic CCAs administration had significantly increased TL2 values compared to the saline-treated mice [*F*(4,35) = 2.727, *P* = 0.0447]. Additionally, post hoc Tukey’s test revealed a statistically significant effect (*P* < 0.05; Table 5), indicating that only verapamil (5 mg/kg), at this dose used, impaired this stage of memory and learning processes after subchronic administration.

**Table 4:** Effects of chronic CCAs or saline administration on the transfer latency to the enclosed arm in the acquisition trial using the mEPM test in mice

Compound	TL2
Saline	22.00 ± 3.50
Verapamil 2.5 mg/kg	32.37 ± 7.02
Verapamil 5 mg/kg	47.25 ± 5.57*
Amlodipine 2.5 mg/kg	36.25 ± 6.22
Amlodipine 5 mg/kg	26.75 ± 5.45

Verapamil (2.5 and 5 mg/kg, i.p.), amlodipine (2.5 and 5 mg/kg), or saline were administered for 13 days. On the 14th day, the mice were injected with CCAs or saline 30 min before the first trial; *n* = 8–10; the data are shown as the means ± SEM

*P < 0.05 vs. saline-treated group (Tukey’s test)

**Table 5:** Effects of chronic CCAs or saline administration on the transfer latency to the enclosed arm in the consolidation trial using the mEPM test in mice

Compound	TL2
Saline	26.62 ± 5.32
Verapamil 2.5 mg/kg	48.00 ± 7.46
Verapamil 5 mg/kg	55.87 ± 10.31*
Amlodipine 2.5 mg/kg	40.87 ± 8.53
Amlodipine 5 mg/kg	27.62 ± 5.83

Verapamil (2.5 and 5 mg/kg, i.p.), amlodipine (2.5 and 5 mg/kg), or saline were administered for 13 days. On the 14th day, the mice were injected with CCAs or saline 30 min immediately after the first trial; *n* = 8–10; the data are shown as the means ± SEM

*P < 0.05 vs. saline-treated group (Tukey’s test)

### Influence of chronic CCAs administration on memory-related responses induced by acute nicotine using the mEPM model in mice

**Fig. 2:**
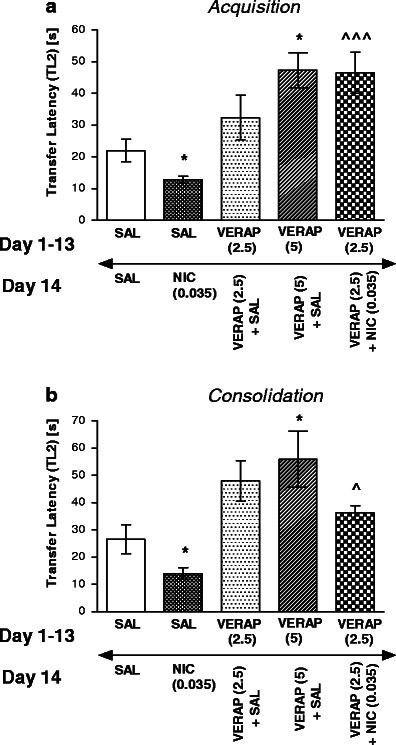
Influence of verapamil administered chronically on the memory-related responses induced by an acute nicotine administration in the acquisition trial (**a**) or consolidation trial (**b**) using the mEPM test in mice. Verapamil (2.5 and 5 mg/kg, i.p.) or saline was administered for 13 days. On the 14th day, the mice were injected with verapamil (2.5 mg/kg) or saline 15 min prior to saline or nicotine (0.035 mg/kg, s.c.) injection, 30 min before the first trial (**a**) or immediately after the first trial (**b**); *n* = 8–10; the data are shown as the means ± SEM; ^*P* < 0.05; ^^^*P* < 0.001 vs. saline-treated and nicotine-challenged group; *P < 0.05 vs. saline-treated group (Tukey’s test)

Figure 2a shows the effect of chronic verapamil pretreatment (2.5 mg/kg) on the memory improvement induced by acute nicotine injection. For memory acquisition during the retention trial, one-way ANOVA revealed a statistically significant effect on TL2 values [*F*(4,38) = 9.406, *P* < 0.0001]. In this experiment, verapamil at the subthresold, ineffective dose, significantly reversed the improvement of memory provoked by an acute injection of nicotine (0.035 mg/kg), thus resulting in an increased TL2 time compared with saline-treated and nicotine challenged group (*P* < 0.001, Tukey’s test). Likewise, for memory consolidation during the retention trial, one-way ANOVA revealed statistically significant effect [*F*(4,39) = 8.229, *P* < 0.0001)]. Indeed, post hoc Tukey’s test revealed that verapamil, at this subthresold, ineffective dose, significantly reversed the improvement of memory provoked by an acute injection of nicotine, thus resulting in an increased TL2 time compared with saline-treated and nicotine challenged group (*P* < 0.05; Fig. 2b).

**Fig. 3:**
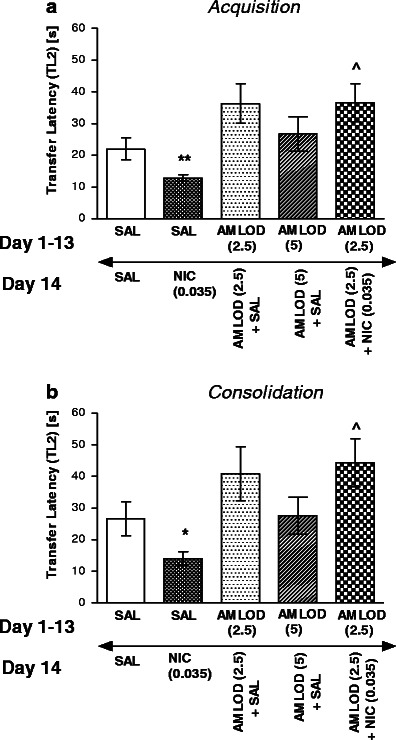
Influence of amlodipine administered chronically on the memory-related responses induced by an acute nicotine administration in the acquisition trial (**a**) or consolidation trial (**b**) using the mEPM test in mice. Amlodipine (2.5 and 5 mg/kg, i.p.) or saline were administered for 13 days. On the 14th day, the mice were injected with amlodipine (2.5 mg/kg) or saline 15 min prior to saline or nicotine (0.035 mg/kg, s.c.) injection, 30 min before the first trial (**a**) or immediately after the first trial (**b**); *n* = 8–10; the data are shown as the means ± SEM; ^*P* < 0.05 vs. saline-treated and nicotine-challenged group; *P < 0.05; **P < 0.01 vs. saline-treated group (Tukey’s test)

In turn, Fig. 3a shows the effect of chronic amlodipine pretreatment (2.5 mg/kg) on the memory improvement induced by acute nicotine injection. For memory acquisition during the retention trial, one-way ANOVA revealed a statistically significant effect on TL2 values [*F*(4,37) = 5.052; *P* < 0.0024]. In this experiment, amlodipine at this ineffective dose significantly reversed the improvement of memory provoked by an acute injection of nicotine (0.035 mg/kg), thus resulting in an increased TL2 time compared with saline-treated and nicotine challenged group (*P* < 0.05, Tukey’s test). Similarly, for memory consolidation during the retention trial, one-way ANOVA revealed that there was statistically significant effect [*F*(4,37) = 4.391; *P* = 0.0053)]. Indeed, post hoc Tukey’s test revealed that amlodipine, at the ineffective dose of 2.5 mg/kg, significantly reversed the improvement of memory provoked by an acute injection of nicotine, thus resulting in an increased TL2 time compared with saline-treated and nicotine challenged group (*P* < 0.05; Fig. 3b).

## Discussion

In the present study, we intended to investigate the effect of nicotine on memory-related behavior as well as the influence of acute and/or subchronic administration of different CCAs, at the large range of doses, on the acquisition and consolidation of memory-related processes and the procognitive effects of nicotine using the recently developed mEPM memory model in mice. Our data extend present knowledge about the influence of pharmacological blockade of L-type VDCC on memory and learning processes in the context of possible interactions with cholinergic transmission.

It has been well documented that nicotine, an alkaloid present in tobacco, is responsible for pharmacological actions of smoking and for its addictive effects, including drug-seeking and relapse despite harmful effect (Dani and De Biasi 2001; Di Chiara 2000; Zaniewska et al. 2009). Nicotine action is due to the activation of different types of the nAChRs in the midbrain dopaminergic reward pathway, especially in the ventral tegmental area (VTA) (Maskos et al. 2005; Stolerman and Shoaib 1991). Moreover, nicotine, as the prototypical nonselective agonist for nAChRs, has long been known to possess procognitive activity in human smokers and nonsmokers (Levin 2002; Levin and Rezvani 2000). The above-mentioned property of nicotine may be of therapeutic benefit in chronic disease states, such as AD, Parkinson’s disease, schizophrenia, or attention-deficit/hyperactivity disorder (Bencherif and Schmitt 2002; Buccafusco 2004; Leonard and Bertrand 2001; Newhouse et al. 1997). However, the side-effect profile of nicotine, e.g., its addictive and psychomotor stimulant properties, precludes the clinical use of this compound or of its analogs.

In experimental animals, nicotine has been found to improve cognitive behavior, although other studies have not found improvements; on the contrary, some have found memory impairments (Biala and Kruk 2008; Hefco et al. 2003; Levin et al. 2006; Puma et al. 1999; Rezvani and Levin 1998). Some of the differences in findings appear to be due to the dose used or the type of cognitive model. Experiments emphasizing nicotine ability to enhance memory and learning, including acquisition, consolidation, and retention, have been performed, both in laboratory animals and humans, and it seems to result from activation of central nAChRs in the prefrontal cortex, hippocampus, and amygdala (Levin and Rezvani 2000; Levin and Simon 1998). Activation of these receptors provokes the release of several neurotransmitters, including dopamine, noradrenaline, 5-HT, ACh, gamma-aminobutyric acid, glutamate, and histamine (Wonnacott 1997), important in the regulation of memory processes. Many of nicotine-related behavioral effects, including memory responses, can be characterized by a dose-dependent inverted U-shaped correlation, as confirmed in our study. It means that, when a particular dose of nicotine is exceed, nicotine-induced responses diminish rapidly. It is specific to the higher nicotine doses, which induce little neuronal activation in many neuronal structures, e.g., the NAC, amygdala, and other limbic areas such as the septum, hippocampus, and hypothalamus. Responsible for this nicotine biphasic pattern of action is acute nicotine tolerance and related desensitization of nicotinic receptors (Wang and Sun 2005).

As noted above, nicotine is a nonselective agonist for all nAChR subtypes, but two subtypes mainly expressed in the brain are of major interest in this context: the heteromeric α4β2, α3β4 and the homomeric α7 (Marubio et al. 2003; Maskos et al. 2005). Evidence from neuroanatomical, electrophysiological, and behavioral studies supports a role for these receptor subtypes in drug dependence and in processes of learning and memory. The α7 subtype, because of its high density in the hippocampus, the amygdale, or the prefrontal cortex, seems to be strongly implicated in cognition (Buccafusco 2004; Levin and Simon 1998; Paterson and Nordberg 2000), as among the brain structures, these areas in particular have been revealed to be important for memory regulation. In this context, the results suggest that synaptic plasticity [i.e., long term potentiation (LTP) of glutamatergic inputs] in the VTA underlies the influence of nicotine on dopamine release in the nucleus accumbens and prefrontal cortex, and this type of LTP may contribute to the effects of nicotine on both learning and memory or addiction (Mansvelder and McGehee 2000, 2002). Determining the basic roles of nicotinic receptor subtypes throughout the central nervous system for various aspects of cognitive processes could facilitate nAChR-related therapeutic drug development.

Concerning calcium homeostasis, it is well established that CCAs are a heterogeneous group of drugs, which have been subdivided into three classes based on chemical structure, pharmacokinetic profile, and therapeutic use: the dihydropyridines (e.g., nimodipine, nicardipine, nifedipine), the benzothiazapines (e.g., diltiazem), and the phenylalkylamines (e.g., verapamil). Drugs in all three classes block calcium entry through specific L-type channels in vascular and cardiac smooth muscle and on neuronal cell bodies (Bean 1989; Miller 1987). All of the CCAs are potent vasodilators, and their functional effect provides the rationale for their use in the treatment of various cardiovascular and neurological conditions, such as hypertension, cerebrovascular disease, arrhythmias, and migraine (Triggle 1999). However, little data are available about the cognitive effects of CCAs, as only some dihydropyridine derivatives were revealed to facilitate memory from several types of learning in adult animals improving retention and/or acquisition of memory-related processes (Genkova-Papazova et al. 1997; Quartermain et al. 2001). Other studies have also suggested that the CCAs may be useful as general cognitive enhancers on the basis of their ability to improve learning and memory in neurologically normal young adult animals. For instance, peripherally administered CCAs have been shown to reverse experimentally induced amnesias, to ameliorate the effects of brain lesions on learning, or to improve spatial working memory (Finger et al. 1990; Genkova-Papazova et al. 2001; Levy et al. 1991; Zupan et al. 1996). Accordingly, in our study, amlodipine, flunarizine, and nicardipine improved the acquisition of memory, while amlodipine, nimodipine, nifedipine, and verapamil improved the consolidation of memory, after an acute injection. However, the interpretation of these data is complicated by the results of other studies, which have either failed to find any effects of CCAs on learning or retention or, on the contrary, have demonstrated impaired cognition following their administration (Isaacson et al. 1989; Maurice et al. 1995; Nikolaev and Kaczmarek 1994). The inconsistency of the findings concerning memory-related effects of CCAs suggests that memory enhancement after their administration in young animals may not be a robust phenomenon and also indicates that the variables, which determine their effect on learning and memory processes, have yet to be completely identified. Among the factors that may determine these effects, one can propose route of administration, i.e., central vs. peripheral, dosing schedule, i.e., acute vs. chronic administration, time of treatment, i.e., pre-training vs. post-training, as well as other variables related to the kind of behavioral tasks used to evaluate memory in animals. Indeed, the notion that blockade of L-type VDCC can improve learning and memory appears paradoxical since it is widely accepted that calcium influx into neurons is a major triggering event and the first step in the cascade of biochemical reactions, which underlies memory acquisition and storage (Izquierdo and Medina 1997; Nicoll and Malenka 1995). Previous experiments have hypothesized various explanations for that paradoxical enhancement of learning by L-type VDCC blockers, including compensatory cellular changes, or their nonspecific vasodilatory effects (Quevedo et al. 1998; Vetulani et al. 1997). Importantly, this learning enhancement has been observed repeatedly to disappear as the dose of L-type VDCC antagonists increases, suggesting that it results from modulation rather than complete blockade of the channels.

In general, important role of L-type VDCC in learning and memory is due to their involvement on the synaptic plasticity (i.e., the long-lasting LTP) of hippocampal dentate CA1 field, which considers being one possible cellular mechanism underlying cognition (Bliss and Collingridge 1993; Lashgari et al. 2006). In vivo and in vitro electrophysiological recordings of hippocampal neuron function have demonstrated the presence of L-type VDCCs, and activity-dependent Ca^2+^ entry into neurons to initiate LTP in this region has been described (Freir and Herron 2003; Morgan and Teyler 1999) The calcium entry into the neurons that induces this phenomenon can occur when *N*-methyl-d-aspartate (NMDA) receptors and VDCCs are activated (Woodside et al. 2004), but the formation of short-term memories seems to be dependent upon activation of NMDA receptors, while activation of L-type VDCCs is required for long-term memory formation (Borroni et al. 2000).

As recent electrophysiological study revealed the strong affinity of amlodipine and verapamil to the nAChRs (Jozwiak et al. 2005), the purpose of the present experiment was also to attempt the memory-enhancing capability of these two CCAs, using chronic, rather than acute, drug administration. Relatively little preclinical research has investigated the effects of chronic administration of potential cognitive enhancers/inhibitors, and it is therefore uncertain whether drugs, which influence memory when administered acutely, will, in all instances, continue to do so under conditions of chronic administration. Our results showed that, contrary to an acute administration, only verapamil, but not amlodipine, after repetitive injections, impaired both acquisition and consolidation of memory processes in mice. Verapamil is an L-type VDCC blocker of the phenylalkylamine group, which has no major side effects shared by other CCAs. There are controversial reports about cognitive effect of chronic and acute administration of verapamil using different memory tests (Lashgari et al. 2006). Previous studies demonstrated that, after an acute or chronic injection, verapamil has no effect on acquisition of passive avoidance and other learning tasks (Borroni et al. 2000; Cain et al. 2002), but other studies have reported an impairment in long-term memory after chronic verapamil administration in the eight-arm radial maze task (Woodside et al. 2004), similarly to our results. Moreover, central injection of verapamil into the hippocampal dentate gyrus and amygdala impairs memory retention and LTP induction in different learning tasks (Freir and Herron 2003; Bauer et al. 2002). On the contrary, some investigators have described a facilitatory role for verapamil in memory retrieval by linear maze learning task in mice (Quartermain et al. 2001). Other possible mechanisms of cognitive effects of verapamil can also underlie its different effects as this CCA can also act as a potent antagonist of 5-HT_2_ receptors, interacts with both α1 and α2 adrenoceptors, and has also been shown to inhibit reuptake of 5-HT, dopamine, and noradrenaline into rat forebrain synaptosomes (Borroni et al. 2000). As for amlodipine, this compound is known to bind directly to the α-subunit of the L-type VDCC channel complex (Quartermain 2000; Quartermain et al. 1993), but also this CCA has been shown to inhibit free radical-induced damage to cell membrane independent of channel blockade (Mason et al. 1999), suggesting as another possibility that these properties might underlie the effect of amlodipine on memory enhancement after an acute injection (Paris et al. 2011), as confirmed by our work.

Concerning the molecular mechanisms of CCAs/nicotine interactions measured in the present work, increasing evidence suggests that changes in calcium channel function play an essential role in nicotine tolerance and dependence development. Not surprisingly, L-type VDCC antagonists have been shown, also in our recent works, to attenuate the signs of nicotine, physical dependence in animals (Biala and Weglinska 2005), as well as other behavioral effects of nicotine, including its reinforcing and stimulant effects, like the acquisition and the expression of nicotine-induced sensitization and place preference including reinstatement phenomenon (Biala 2003; Biala and Budzynska 2006, 2008; Biala and Weglinska 2004). Our present results revealed that flunarizine, nicardipine, amlodipine, verapamil, and bupropion, considered to be a noncompetitive nicotinic-receptor antagonist at α3β2, α4β2, and at α3β4 ganglionic-type of nicotinic receptors (Slemmer et al. 2000), all at the subthresold doses, significantly reversed the nicotine-induced improvement of memory acquisition, while flunarizine, verapamil, and bupropion, but not nicardipine or amlodipine, significantly reversed the improvement of memory consolidation provoked by an acute injection of nicotine, thus resulting in an increased TL2 time compared with nicotine-treated group. Moreover, chronic pretreatment of both verapamil and amlodipine, at the subthresold, ineffective dose, significantly reversed the improvement of both acquisition and consolidation of memory provoked by an acute injection of nicotine, thus resulting in an increased TL2 time compared with saline-treated and nicotine-challenged group. However, the interpretation of these data and the observed difference in inhibitory actions on nicotine-induced cognitive behaviors among CCAs is complicated by other results of different studies in humans and animals already mentioned, which have either failed to find any effects of CCAs on cognition or, on the contrary, have demonstrated impaired cognition following their administration. Thus, the effect of CCAs chosen for our experiments to deteriorate pathological conditions in AD patients has not been confirmed in all above-mentioned studies.

Previous studies with neuronal preparations have shown that significant amounts of Ca^2+^ enter the neuron following activation of nAChRs, causing a rise in Ca^2+^ concentration (Barrantes et al. 1995), and this effect is sufficient to activate calcium-dependent protein kinases, like, e.g., calcium-dependent calmodulin protein kinase II (CaMKII; Damaj 2000). Furthermore, nAChR-mediated depolarization can activate VDCCs, and this effect potentiates the primary Ca^2+^ signals generated by nicotinic receptor activation (Dajas-Bailador and Wonnacott 2004). It is almost certain that enhanced calcium-dependent CaMKII activity can phosphorylate and modulate functions of other proteins that could play some role in modulating nicotine memory enhancing effects.

From both electrophysiological and behavioral studies, we can better understand the downstream effects of nAChR activation, including activation of VDCCs. As already noted, nicotinic receptors are found throughout the brain where they act presynaptically to increase synaptic efficacy by increasing neurotransmitter release, probably by recruiting calcium channels to engage downstream signaling event. In both the central and peripheral nervous systems, nAChRs are inhibited by several drugs that are commonly considered to be specific for L-type calcium channels. In turn, evidence suggests that activation of postsynaptic nAChRs, also by nicotine by itself, can activate L-type VDCCs engaging downstream signaling mechanisms including the above-mentioned CaMKII pathway, to induce LTP and alter CREB-dependent gene expression (Levin et al. 2006; Wheeler et al. 2006). Moreover, L-type VDCC can also oppose the effects of nAChR activation. CCAs block currents through VDCC either by preventing channel activation or acting as direct pore blockers; however, L-type CCAs modulators are also noncompetitive inhibitors at nAChRs by slow open-channel blockade and/or by promoting nAChR desensitization (Adam and Henderson 1990; Buisson and Bertrand 1998; Jozwiak et al. 2005), as can be confirmed by the present research.

## Conclusion

To sum up, our data indicate that nicotine and CCAs chosen were capable of producing significant improvement in memory effect after an acute injection in the mEPM test in mice. Moreover, we revealed that both bupropion, a nAChR antagonist, and CCAs attenuated nicotine-induced improvement in memory acquisition and/or consolidation. Our results further established that interactions of L-type VDCC antagonists with nAChRs can have a major impact on cellular events downstream of nAChR activation by nicotine. However, the role of CCAs interactions with nAChRs in signaling has not been examined in detail yet. The present findings are the first to demonstrate that, at concentrations typically used to block L-type VDCC, these antagonists can modify (i.e., improve after an acute administration or decrease after chronic injection) memory-related processes, and can block the memory enhancing effects of nicotine by inhibiting nAChR currents and downstream signaling. As such, it is possible to speculate on the influence of calcium homeostasis on the adaptive changes underlying the cognitive effects of nicotine, but further research is necessary in order to determine their efficacy and safety.

## References

[CR1] Adam LP, Henderson EG (1990). Calcium channel effectors are potent non-competitive blockers of acetylcholine receptors. Pflugers Arch.

[CR2] Barrantes GE, Murphy CT, Westwick J, Wonnacott S (1995). Nicotine increases intracellular calcium, in rat hippocampal neurons via voltage-gated calcium channels. Neurosci Lett.

[CR3] Bauer EP, Schaffe GE, LeDoux JE (2002). NMDA receptors and L-type voltage-gated calcium channels contribute to long-term potentiation and different components of fear memory in the lateral amygdale. J Neurosci.

[CR4] Bean BP (1989). Classes of calcium channels in vertebrate cells. Annu Rev Physiol.

[CR5] Bencherif M, Schmitt JD (2002). Targeting neuronal nicotinic receptors: a path to new therapies. Current Drug Targets CNS Neurol Disord.

[CR6] Biala G (2003). Calcium channel antagonists suppress nicotine-induced place preference and locomotor sensitization in rodents. Pol J Pharmacol.

[CR7] Biala G, Budzynska B (2006). Reinstatement of nicotine-conditioned place preference by drug priming: effects of calcium channel antagonists. Eur J Pharmacol.

[CR8] Biala G, Budzynska B (2008). Calcium-dependent mechanisms of the reinstatement of nicotine conditioned place preference by drug priming in rats. Pharmacol Biochem Behav.

[CR9] Biala G, Weglinska B (2004). Calcium channel antagonists attenuate cross-sensitization to the rewarding and/or locomotor effects of nicotine, morphine and MK-801. J Pharm Pharmacol.

[CR10] Biala G, Weglinska B (2005). Blockade of the expression of mecamylamine-precipitated nicotine withdrawal by calcium channel antagonists. Pharmacol Res.

[CR11] Biala G, Kruk M (2008). Cannabinoid receptor ligands suppress memory-related effects of nicotine in the elevated plus maze in mice. Behav Brain Res.

[CR12] Bliss TV, Collingridge GL (1993). A synaptic model of memory: long term potentiation in the hippocampus. Nature.

[CR13] Borroni AM, Fichtenholtz H, Woodside BL, Teyler TJ (2000). Role of voltage-dependent calcium channel long-term potentiation (LTP) and NMDA LTP in spatial memory. J Neurosci.

[CR14] Buccafusco JJ (2004). Neuronal nicotinic receptor subtypes: defining therapeutic targets. Mol Intervent.

[CR15] Buisson B, Bertrand D (1998). Allosteric modulation of neuronal nicotinic acetylcholine receptors. J Physiol Paris.

[CR16] Cain CK, Blouin AM, Barad M (2002). L-type voltage-gated calcium channels are required for extinction, but not for acquisition or expression, of conditional fear in mice. J Neurosci.

[CR17] Dajas-Bailador F, Wonnacott S (2004). Nicotinic acetylcholine receptors and the regulation of neuronal signalling. Trends Pharmacol Sci.

[CR18] Damaj MI (2000). The involvement of spinal Ca(2+)/calmodulin-protein kinase II in nicotine-induced antinociception in mice. Eur J Pharmacol.

[CR19] Dani JA, De Biasi M (2001). Cellular mechanisms of nicotine addiction. Pharmacol Biochem Behav.

[CR20] Di Chiara G (2000). Role of dopamine in the behavioural actions of nicotine related to addiction. Eur J Pharmacol.

[CR21] Finger S, Green L, Tarnoff ME, Mortman KD, Andersen A (1990). Nimodipine enhances new learning after hippocampal damage. Exp Neurol.

[CR22] Freir DB, Herron CE (2003). Inhibition of L-type voltage dependent calcium channels causes impairment of long-term potentiation in the hippocampal CA1 region in vivo. Brain Res.

[CR23] Friederich P, Dybek A, Urban BW (2000). Stereospecific interaction of ketamine with nicotinic acetylcholine receptors in human sympathetic ganglion-like SH-SY5Y cells. Anesthesiology.

[CR24] Genkova-Papazova MG, Petkova BP, Lazarova-Bakarova M, Boyanova E, Staneva-Stoytcheva D (1997). Effects of flunarazine and nitrendipine on electroconvulsive shock- and clonidine-induced amnesia. Pharmacol Biochem Behav.

[CR25] Genkova-Papazova MG, Petkova BP, Shishkova N, Lazarova-Bakarova M (2001). Effect of the calcium channel blockers nifedipine and diltiazem on pentylenetetrazole kindling-provoked amnesia in rats. Eur Neuropsychopharmacol.

[CR26] Gotti C, Zoli M, Clementi F (2006). Brain nicotinic acetylcholine receptors: native subtypes and their relevance. Trends Pharmacol Sci.

[CR27] Hefco V, Yamada K, Hefco A, Hritcu L, Tiron A, Olariu A, Nabeshima T (2003). Effects of nicotine on memory impairment induced by blockade of muscarinic, nicotinic and dopamine D2 receptors in rats. Eur J Pharmacol.

[CR28] Hliňák Z, Krejčí I (1998). Concurrent administration of subeffective doses of scopolamine and MK-801 produces a short-term amnesia for the elevated plus-maze in mice. Behav Brain Res.

[CR29] Hliňák Z, Krejčí I (2002). Oxiracetam prevented the scopolamine but not the diazepam induced memory deficits in mice. Behav Brain Res.

[CR30] Isaacson RL, Maier DL, Mandel AH (1989). Post training or pretest administration of nimodipine fails to affect retention of a simple learned association. Physiol Behav.

[CR31] Itoh J, Nabeshima T, Kameyama T (1990). Utility of an elevated plus maze for the evaluation of memory in mice: effects of nootropics, scopolamine and electroconvulsive shock. Psychopharmacology.

[CR32] Itoh J, Nabeshima T, Kameyama T (1991). Utility of an elevated plus-maze for dissociation of amnesic and behavioral effects of drugs in mice. Eur J Pharmacol.

[CR33] Izquierdo I, Medina JH (1997). The biochemistry of memory formation and its regulation by hormones and neuromodulators. Psychobiology.

[CR34] Jackson KJ, Martin BP, Changeux JP, Damaj MI (2008). Differential role of nicotinic acetylcholine receptor subunits in physical and affective withdrawal signs. J Pharmacol Exp Ther.

[CR35] Jozwiak K, Moaddel R, Ravichandran S, Plazinska A, Kozak J, Patel S (2008). Exploring enantiospecific ligand-protein interactions using cellular membrane affinity chromatography: chiral recognition as a dynamic process. J Chromatogr B Analyt Technol Biomed Life Sci.

[CR36] Jozwiak K, Moaddel R, Yamaguchi R, Ravichandran S, Collins JR, Wainer IW (2005). Qualitative assessment of IC50 values of inhibitors of the neuronal nicotinic acetylcholine receptor using a single chromatographic experiment and multivariate cluster analysis. J Chromatogr B Analyt Technol Biomed Life Sci.

[CR37] Khachaturian AS, Zandi PP, Lyketsos CG, Hayden KM, Skoog I, Norton MC (2006). Antihypertensive medication use and incident Alzheimer disease. The Cache County Study. Arch Neurol.

[CR38] Kruk M, Tendera K, Biala G (2011). Memory-related effects of cholinergic receptor ligands in mice as measured by the elevated plus maze test. Pharmacol Rep.

[CR39] Kruk-Slomka M, Budzynska B, Biala G (2012). Involvement of cholinergic receptors in the different stages of memory measured in the modified elevated plus maze test in mice. Pharmacol Rep.

[CR40] Lashgari R, Motamedi F, Asl SZ, Shahidi S, Komaki A (2006). Behavioral and electrophysiological studies of chronic oral administration of L-type calcium channel blocker verapamil on learning and memory in rats. Behav Brain Res.

[CR41] Leonard S, Bertrand D (2001). Neuronal nicotinic receptors: from structure to function. Nicotine Tobacco Res.

[CR42] Levin ED (2002). Nicotinic receptor subtypes and cognitive function. J Neurobiol.

[CR43] Levin ED, McClernon FJ, Rezvani AH (2006). Nicotinic effects on cognitive function: behavioral characterization, pharmacological specification and anatomic localization. Psychopharmacology.

[CR44] Levin ED, Rezvani AH (2000). Development of nicotinic drug therapy for cognitive disorders. Eur Pharmacol.

[CR45] Levin ED, Simon BB (1998). Nicotinic acetylcholine involvement in cognitive function in animals. Psychopharmacology.

[CR46] Levy A, Kong RM, Stillman MJ, Shukitt-Hale B, Kadar T, Rauch T, Lieberman HR (1991). Nimodipine improves spatial working memory and elevates hippocampal acetylcholine in young rats. Pharmacol Biochem Behav.

[CR47] Mansvelder HD, McGehee DS (2000). Long-term potentiation of excitatory inputs to brain reward areas by nicotine. Neuron.

[CR48] Mansvelder HD, McGehee DS (2002). Cellular and synaptic mechanisms of nicotine addiction. J Neurobiol.

[CR49] Marubio LM, Gardier AM, Durier S, David D, Klink R, Arroyo- Jimenez MM (2003). Effects of nicotine in the dopaminergic system of mice lacking the alpha 4 subunit of neuronal nicotinic acetylcholine receptors. Eur J Neurosci.

[CR50] Maskos U, Molles BE, Pons S, Besson M, Guiard BP, Guilloux JP (2005). Nicotine reinforcement and cognition restored by targeted expression of nicotinic receptors. Nature.

[CR51] Mason PR, Walter MF, Trumbore MW, Olmstead EG, Mason PE (1999). Membrane antioxidant effects of the charged dihydropyridine calcium antagonist amlodipine. J Mol Cell Cardiol.

[CR52] Maurice T, Bayle J, Privat A (1995). Learning impairment following acute administration of the calcium channel antagonist nimodipine in mice. Behav Pharmacol.

[CR53] Maxwell CJ, Hogan DB, Ebly EM (1999). Calcium-channel blockers and cognitive function in elderly people: results from the Canadian Study of Health and Aging. CMAJ.

[CR54] Miller RJ (1987). Multiple calcium channels and neuronal function. Science.

[CR55] Moaddel R, Jozwiak K, Wainer IW (2007). Allosteric modifiers of neuronal nicotinic acetylcholine receptors: new methods, new opportunities. Med Res Rev.

[CR56] Morgan SL, Teyler TJ (1999). VDCCs and NMDARs underlie two forms of LTP in CA1 hippocampus in vivo. J Neurophysiol.

[CR57] Newhouse PA, Potter A, Levin ED (1997). Nicotinic system involvement in Alzheimer’s and Parkinson’s diseases. Implications for therapeutics. Drugs Aging.

[CR58] Nicoll RA, Malenka RC (1995). Contrasting properties of two forms of long term potentiation in the hippocampus. Nature.

[CR59] Nikolaev E, Kaczmarek L (1994). Disruption of two-way active avoidance behavior by nimodipine. Pharmacol Biochem Behav.

[CR60] Paris D, Bachmeier C, Patel N, Quadros A, Volmar C-H, Laporte V (2011). Selective antihypertensive dihydropyridines lower Aβ accumulation by targeting both the production and the clearance of Aβ across the blood–brain barrier. Mol Med.

[CR61] Paterson D, Nordberg A (2000). Neuronal nicotinic receptors in the human brain. Prog Neurobiol.

[CR62] Picciotto MR, Caldarone BJ, King SL, Zachariou V (2000). Nicotinic receptors in the brain. Links between molecular biology and behavior. Neuropsychopharmacology.

[CR63] Picciotto MR, Zoli M (2002). Nicotinic receptors in aging and dementia. J Neurobiol.

[CR64] Puma C, Deschaux O, Molimard R, Bizot JC (1999). Nicotine improves memory in an object recognition task in rats. Eur Neuropsychopharmacol.

[CR65] Quartermain D (2000). Chronic administration of the Ca^2+^ channel blocker amlodipine facilitates learning and memory in mice. Eur J Pharmacol.

[CR66] Quartermain D, Garcia DeSoria V, Kwan A (2001). Calcium channel antagonists enhance retention of passive avoidance and maze learning in mice. Neurobiol Learn Memory.

[CR67] Quartermain D, Hawxhurst A, Ermita B, Puente J (1993). Effect of the calcium channel blocker amlodipine on memory in mice. Behav Neural Biol.

[CR68] Quevedo J, Vianna M, Daroit D, Born AG, Kuyven CR, Roesler R, Quillfeldt JA (1998). L type voltage-dependent calcium channel blocker nifedipine enhances memory retention when infused into the hippocampus. Neurobiol Learn Mem.

[CR69] Rezvani AH, Levin ED (1998). Cognitive effects of nicotine. Biol Psychiat.

[CR70] Sabbagh MN, Lukas RJ, Sparks DL, Reid RT (2002). The nicotinic acetylcholine receptor, smoking, and Alzheimer's disease. J Alzheimers Dis.

[CR71] Sharma AC, Kulkarni SK (1992). Evaluation of learning and memory mechanisms employing elevated plus-maze in rats and mice. Prog Neuropsychopharmacol Biol Psychiatry.

[CR72] Slemmer JE, Martin BR, Damaj MI (2000). Bupropion is a nicotinic antagonist. J Pharmacol Exp Ther.

[CR73] Stolerman IP, Shoaib M (1991). The neurobiology of tobacco addiction. Trends Pharmacol Sci.

[CR74] Triggle DJ (1999). The pharmacology of ion channels: with particular reference to voltage-gated Ca^2+^ channels. Eur J Pharmacol.

[CR75] Vetulani J, Battaglia M, Sansone M (1997). Nimodipine on shuttle box avoidance learning in mice: no impairment but slight improvement. Pharmacol Biochem Behav.

[CR76] Wang H, Sun X (2005). Desensitized nicotinic receptors in brain. Brain Res Rev.

[CR77] Wheeler DG, Barrett CF, Tsien RW (2006). L-type calcium channel ligands block nicotine-induced signaling to CREB by inhibiting nicotinic receptors. Neuropharmacology.

[CR78] Wonnacott S (1997). Presynaptic nicotinic ACh receptors. Trends Neurosci.

[CR79] Woodside BL, Borroni AM, Hammonds MD, Teyler TJ (2004). NMDA receptors and voltage-dependent calcium channels mediate different aspects of acquisition and retention of a spatial memory task. Neurobiol Learn Memory.

[CR80] Zaniewska M, Przegalinski E, Filip M (2009). Nicotine dependence—human and animal studies, current pharmacotherapies and future perspectives. Pharmacol Rep.

[CR81] Zupan G, Vitezic D, Mrsic J, Matesic D, Simonic A (1996). Effects of nimodipine, felodipine and amlodipine on electroconvulsive shock-induced amnesia in the rat. Eur J Pharmacol.

